# Influence of Multiple rISC Channels on the Maximum Efficiency and Roll-Off of TADF OLEDs

**DOI:** 10.1021/acs.jpcc.4c02993

**Published:** 2024-09-19

**Authors:** Paloma Lays dos Santos, Daniel de Sa Pereira, Chan Seok Oh, Nadzeya Kukhta, Ha Lim Lee, Jun Yeob Lee, Andrew P. Monkman

**Affiliations:** † Department of Electronic and Electrical Engineering, University of Sheffield, Mappin St, Sheffield S1 3JD, U.K.; ‡ Department of Physics, Durham University, South Road, Durham DH1 3LE, U.K.; § School of Chemical Engineering, Sungkyunkwan University, 2066, Seobu-ro, Jangan-gu, Suwon, Gyeonggi 16419, Korea; ∥ Department of Chemistry, Durham University, South Road, Durham DH1 3LE, U.K.; ⊥ SKKU Institute of Energy Science and Technology, Sungkyunkwan University, 2066, Seobu-ro, Jangan-gu, Suwon, Gyeonggi 16419, Korea

## Abstract

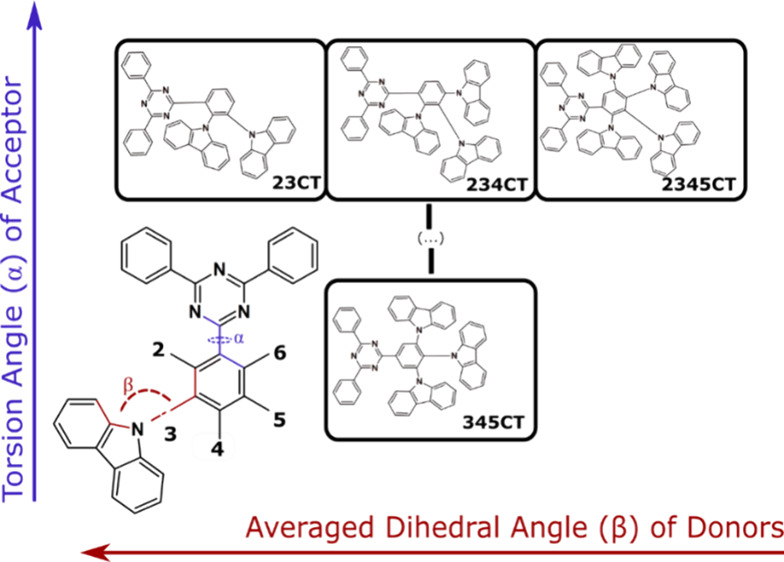

In this work, we look into the detailed photophysical characterization of a multidonor–acceptor (**D-A**) family of thermally activated delayed fluorescent (TADF) emitters to find correlations with their device performance. Increasing the number of closely packed **D**s around the **A** core leads to changes in dihedral angles between **D**s and **A,** affecting the highest occupied molecular orbital (HOMO)/lowest unpccupied molecualar orbital (LUMO) separation and impacting the singlet–triplet energy gaps. Moreover, **D-A** dihedral angles change molecular conjugation affecting the spread of charge-transfer state energies as well as the energy of **D** local triplet states. The coupling between these triplet states and the dispersion in CT states lead to the appearance of multiple rISC channels, a phenomenon that is host-dependent, i.e., hosts with different rigidities twist the dihedral angles differently. We show that different subsets of rISC rates directly impact device performance, where faster rISC leads to external quantum efficiencies above 20% while slower rISC rates act as parasitic traps, severely affecting device roll-off. This explains why emitters with excellent peak external quantum efficiencies can also present very poor roll-off.

## 1 Introduction

Optimizing molecular design strategies for thermally activated delayed fluorescence (TADF) emitters and their performance in organic light-emitting diodes (OLEDs) has been a great challenge when targeting high device efficiencies, stabilities and lifetimes.^1^ This becomes particularly evident when blue TADF-based devices are sought.^2,3^

The basic design strategy of a TADF molecule uses electron-donating (**D**) and electron-accepting (**A**) units bound through an N–C bridge in different proportions and positions (**D-A**, **D-A-D**, **D-A**_**3**_, **D**_**3**_**-A**)^4−10^ that spatially separate and electronically decouple the highest occupied molecular orbital (HOMO) and lowest unoccupied molecular orbital (LUMO).^11^ This yields charge-transfer (^1^CT) singlet and (^3^CT) triplet excited states.^1^ Studies by Monkman and co-workers and Penfold et al. have demonstrated that a local (either **D** or **A**) excited triplet (^3^LE) state can vibronically couple with the ^3^CT, mediating a spin-flip back to ^1^CT.^12−14^ Therefore, very small singlet–triplet energy gaps (Δ*E*_ST_) and vibrational coupling driven spin–orbit coupling are required for efficient reverse intersystem crossing (rISC).

TADF-based OLEDs have already proven to be capable of achieving high external quantum efficiencies (η_ext_), without considering outcoupling effects due to emitter orientation.^15^ However, a problem that these devices suffer from and prevent their use in industrial-level applications is the decrease in η_*ext*_ with the increase of current density, a phenomenon known as efficiency roll-off. This has been revealed to be just as important (or even more important) than the maximum η_ext_ itself. However, understanding of emitter design rules that help suppress this effect has not yet been thoroughly explored. The most obvious explanation behind this effect is the assumption that, if the excited state lifetime of the emitter is too long, the excited states become prone to quenching mechanisms such as triplet–triplet annihilation (TTA) or triplet-polaron quenching (TPQ), especially by charges in a device at high current densities required for high brightness levels,^16,17^ but how long is too long?

Many devices have been reported showing outstanding η_ext_ maximum, such as green TADF devices reaching η_ext_ > 37% by Wu et al.^18^ However, the device roll-off reported shows a strong decline at high current densities.

With this work, our aim is to gain insights into what really controls this phenomenon; is it the photophysics of the emitter, the device optimization, or a combination of both? By introducing new ambipolar host materials, Lin and co-workers make an interesting observation using TADF emitters that show poor photophysical TADF properties and still give high performing devices.^19^ Although these hosts give good charge balance and horizontal orientation which go a long way to explaining good device performance, the roll-off here is still very evident. Thus, charge carrier optimization in the device is very important for high η_ext_ but does not necessarily hinder resistance to roll-off. These results show that whereas device optimization is an important part of the jigsaw puzzle to the “perfect” TADF-OLED device, the complete answer is still unclear.

**Figure 1:**
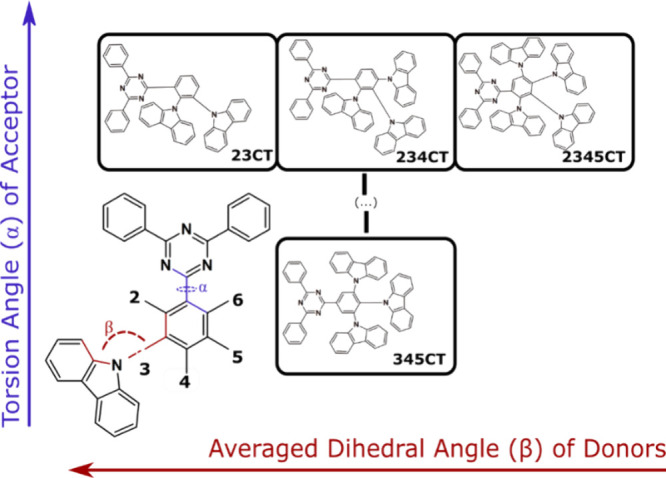
Diagram represents the work involved in this study. Using a molecular design based on a 2,4,6-triphenyl-1,3,5-triazine (TRZ) acceptor (A) and a carbazole (Cz) donor (D), the effect of increasing the number of Ds to the photophysical properties of the emitters is studied. This will affect the dihedral angle (β) between the D and the phenyl ring of the A and the torsional angle (α) between that same phenyl ring and the TRZ core and more importantly, the number of fitted rISC channels and efficiency roll-off in the device. Therefore, four emitters were studied: three of them with *ortho-*substituted Cz and with an increasing number of Ds (from 2 to 3 and 4) and the last with three non-*ortho* closely packed Ds.

To benchmark the understanding that molecular effects have on efficiency roll-off, we must consider emitters from the same family, i.e., using similar **D** and **A** molecules and make a more in-depth study within the photophysics of the molecules themselves. We demonstrated that an effective design route comes from controlling the **D-A** dihedral angle by placing two carbazole (Cz) donors in different positions of the phenyl ring of a triazine acceptor. We were able to see changes in the CT character and conjugation of a set of different **D**_**2**_**-A** emitters, effectively controlling the resulting energy gaps.^20^ Adding a third carbazole **D** unit showed a similar trend - that closely packed **D**s interact sterically and restrict dihedral angles to give better TADF performance.^21^ Device results from these two studies showed a high peak η_ext_ for most materials. However, the resistance to efficiency roll-off varied, in some instances falling very rapidly, a behavior that was not fully understood, making this an ideal family to study such effect. The emitters that showed the best TADF performance from previous studies, having an increasing number of carbazole donor units, are chosen for this study and compared with a novel **D**_**4**_**-A** emitter with four neighboring carbazoles around the **A** (Figure 1).

To address the roll-off conundrum, we compare these four emitters and understand how the number, substitution pattern, and steric interactions of the carbazole substituents affect the TADF mechanism of emitters that already have exceptionally good device performance. We also show how the steric effects of different host environments also impinge on the TADF mechanism and therefore on efficiency roll-off. This is done by understanding the effect of **D** hindrance, proximity of the **D**s to the **A** on the **D-A** dihedral angle and of decoupling due to steric crowding. We elucidate how the smallest differences in twisting within the acceptor (α)–folding around itself with the presence of an -*ortho* carbazole–and the change in dihedral angles of the donors (β) both affect the TADF mechanism and in the roll-off performance of the devices. Notably, we show that more than one rISC channel operates when multiple **D**s are present. This is due to Cz substitutions in different positions interacting with each other, changing dihedral angles and causing different degrees of ^3^LE localization (and energy levels), as well as different overall ^1^CT (^3^CT) energies. This causes two effects: the first is to produce a large heterogeneity and thus dispersion in CT energies and rISC rates; the second is that the nonequivalent ^3^LE states can independently vibronically couple to different states giving rise to more than one rISC channel with different rISC rates. Extending this analysis to the host material, a similar trend is found as host packing similarly affects the **D-A** dihedral angles, producing large effects on rISC, over and above simple polarity effects on CT energies. In devices, the states that lead to slower rISC channels cause poor roll-off even when the primary rISC channel is very efficient. In this way, different roll-off behaviors can be described, for a family of emitters that have high η_ext_.

## 2 Results and Discussion

### 2.1 Molecular Design and DFT Calculation

Carbazole (Cz) and 2,4,6-triphenyl-1,3,5-triazine (TRZ) are two widely used donor (**D**) and acceptor (**A**) units, respectively, for TADF emitters. They have been used in many different combinations and ratios with thoroughly studied photophysical properties.^22−28^ Our previous findings showed that placing 2 and 3 carbazoles around one of the phenyl rings of the triazine affects the dihedral angle of the **D**s.^20,21^ When these **D**s are adjacent to each other and the **A**, the dihedral angle tends to increase toward orthogonality with the **A** unit through **D-D** interactions.^29^ This lowers conjugation, decreases electron exchange energy, and increases the strength of the CT character. In turn, decreased singlet–triplet energy gaps and higher rISC rates are obtained.^20^ To understand further this behavior, we extended the family of materials by synthesizing a **D**_**4**_**-A** emitter, with four carbazoles placed in close proximity to each other (synthetic procedure detailed in **S1**). The result is an emitter with **D**s in the -2/-3/-4/-5 substitution positions around one of the phenyl rings of TRZ **A**, an emitter we named **2345CT**. To fully understand how the number of (carbazole) **D**s affects the photophysics of this family, we compared **2345CT** with three other members: **23CT**, **234CT**, and **345CT**. The latter in particular was chosen^30^ because the carbazoles are placed in close proximity to each other but away from the triazine **A** and thus the effects of the triazine folding (α) can also be understood.

**Figure 2:**
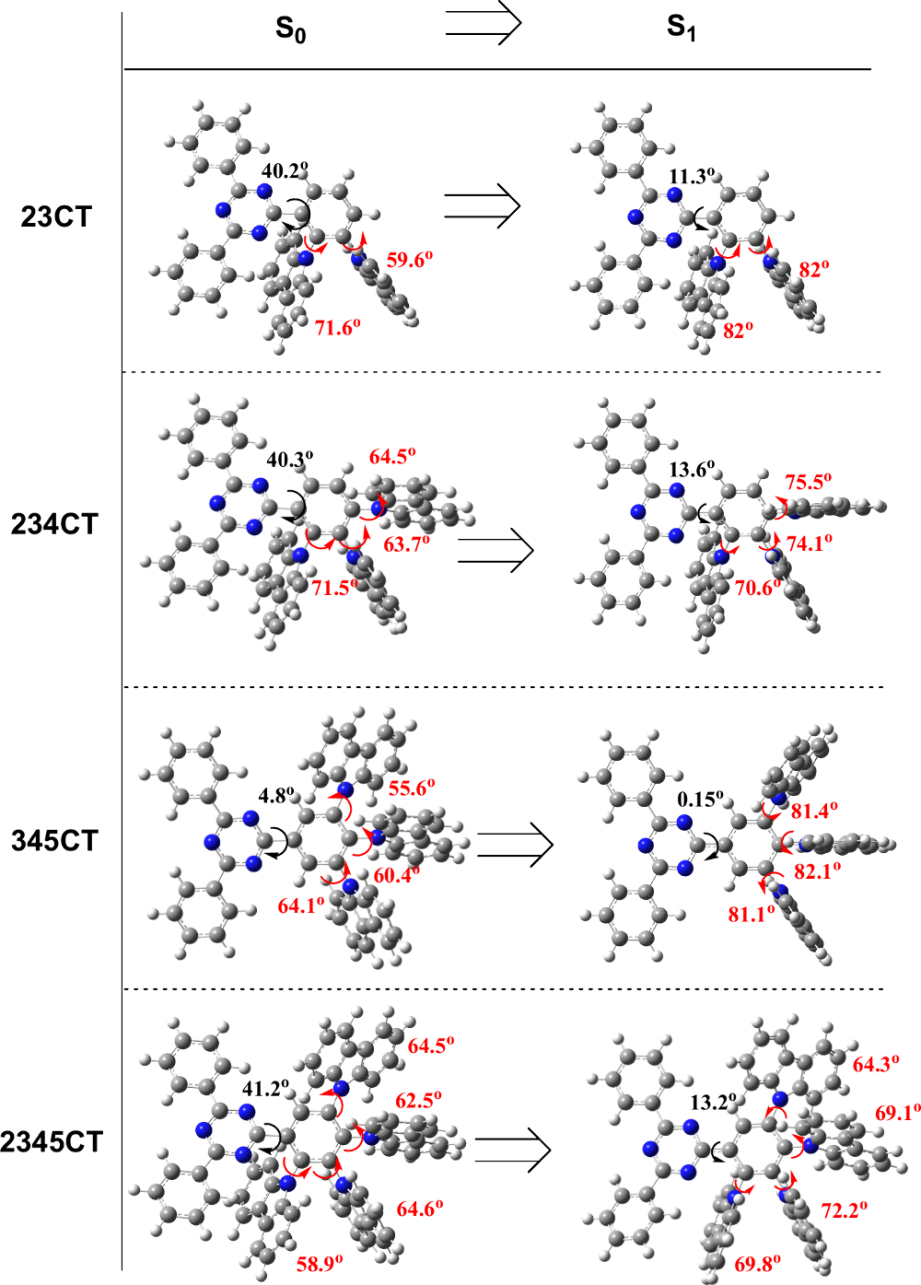
Ground (rCAM-B3LYP/6-31G(d)) and excited (TDA-DFT CAM-B3LYP/6-31G(d)) state geometries of the studied compounds along with the corresponding values of dihedral angles.

Ground state geometries of the studied compounds were accessed at the rCAM-B3LYP/6-31G(d) level of theory (Figure 2), while the S_1_ excited state configurations were obtained using the Tamm-Dancoff approximation at the TDA-DFT CAM-B3LYP/6-31G(d) level. The dihedral angles of each emitter are affected by the substitution position in a 2-fold manner: (i) the linking acceptor phenyl ring becomes distorted from planarity by the close proximity to the carbazole donors, particularly with an -*ortho* substitution and (ii) increasing number of closely packed carbazoles interact sterically, affecting the dihedral angles.^20^ In the ground state, the carbazoles of **23CT** are not equivalent: dihedral angles of the ortho-carbazole is ca. 71.6°, while the other is twisted by 59.6°. In **234CT** the −2 position remains at ca. 71.5° while the −3 and −4 position carbazoles relax to lower dihedrals of ca. 64°. Interestingly, the torsion angle of the ortho-carbazole in **2345CT** is smaller (58.9°) compared to the other two ortho-substituted triazines with the other three donors being almost equivalent (62–64°). Finally, three carbazoles are nonequivalent in **345CT**, with the dihedral angles ranging from 56 to 64°. Clearly, the difference between the ortho-substituted compounds and symmetrical **345CT** lies in the additional twist of the TRZ unit. While the Trz angle reaches 41.2° in the most sterically hindered **2345CT**, the triphenyltriazine unit is almost fully conjugated in **345CT** (Trz angle of 4.8°). The situation changes remarkably in the S_1_ excited state (Figure 2). While the acceptor unit planarizes completely in the symmetrical **345CT**, only partial planarization (Trz angle of ca. 11–13°) is observed in the more sterically crowded ortho-substituted compounds. Noteworthy, the ortho-carbazole in **23CT** is located almost perpendicularly in the S_1_ state, while the introduction of the additional donor units results in a ca. 70 °Cz angle.

Frontier orbital analysis of the four emitters was carried out to obtain the HOMO/LUMO distributions of **2345CT** in comparison to the other three molecules; additional computational results are shown in Figure S2. Overall, the ground state HOMO/LUMO distributions of **23CT**, **234CT** and **2345CT** are similar, with the LUMO being localized at the TRZ acceptor. The HOMO is highly localized within the −2/-3 carbazoles in all three; however, increasing the number of **D**s results in increased orbital distribution into the -*para* substituent carbazoles. Interestingly, in **2345CT**, no distribution of HOMO is seen in the carbazole at −5 position. **234CT** and **2345CT** have therefore almost identical orbital distributions. By contrast, **345CT** shows even HOMO distribution across all **D**s and LUMO mostly localized at the triazine core and phenyl linker. Closely packed **D**s placed in proximity to the **A** force it to bend around itself, pushing the LUMO away from the benzene ring. These small changes and nonequivalent dihedral angles are very important because the electron exchange energy and hence rISC rates depend exponentially on the magnitudes of the energy gaps between the coupled singlet and triplet states.^4^ Interestingly, the HOMOs of the ortho-substituted CT derivatives (**23CT**, **234CT** and **2345CT**) in the first excited state share identical electron wave function distribution, regardless of the differences in the dihedral angles: only carbazoles attached to 2,3-positions of the benzene ring are involved. In contrast, as **345CT** becomes symmetrical in the S_1_ state, all three donors are involved in HOMO. In turn, LUMO of all the CT derivatives is localized on the 2-phenyl-1,3,5-triazine fragment, with a minor leakage onto the 4-carbazole in the case of **234CT**, **345CT** and **2345CT**. Of note, the leakage is the highest in the case of **345CT** due to the planarized skeleton of the acceptor, leading to the highest HOMO/LUMO overlap within the series. On the contrary, ortho-substituted derivatives display a remarkable frontier orbital decoupling in the excited state.

### 2.2 Photophysics

As is typical of **D-A** type TADF molecules used in OLEDs, the absorption spectra of all 4 materials in a dichloromethane (DCM) solution is a superposition of the respective **D** and **A** absorptions, which give insight into their electronic decoupling, fundamental for charge-transfer (CT); additional photophysical results are shown in Figure S3a. By comparing with the absorption of each individual **D** and **A** unit (inset of Figure S3a), peaking at approximately 275 nm is the π–π* transition of the TRZ **A** unit and transitions between ∼300 and 340 nm are attributed to the π–π* absorption of the carbazole **D** unit. At low energies, a third band appears peaking at 375 nm assigned as “direct” CT absorption with a n-π* (or mixed π–π*/n−π*)^4,31−33^ character.

Emission spectra also show typical features of **D-A** type TADF molecules, characterized by solvatochromism.^34^ The solvatochromic shift of each emission spectrum is shown in Figure S3b. All the molecules containing **Ds** in the -*ortho* position showed similar shifts from methylcyclohexane (MCH) to toluene, however, **234CT** and **2345CT** have a bigger redshift in DCM. **345CT**, with no -*ortho* substitution, showed the highest bathochromic shift, in line with having the smaller **D-A** dihedral angles.^20^

**Figure 3:**
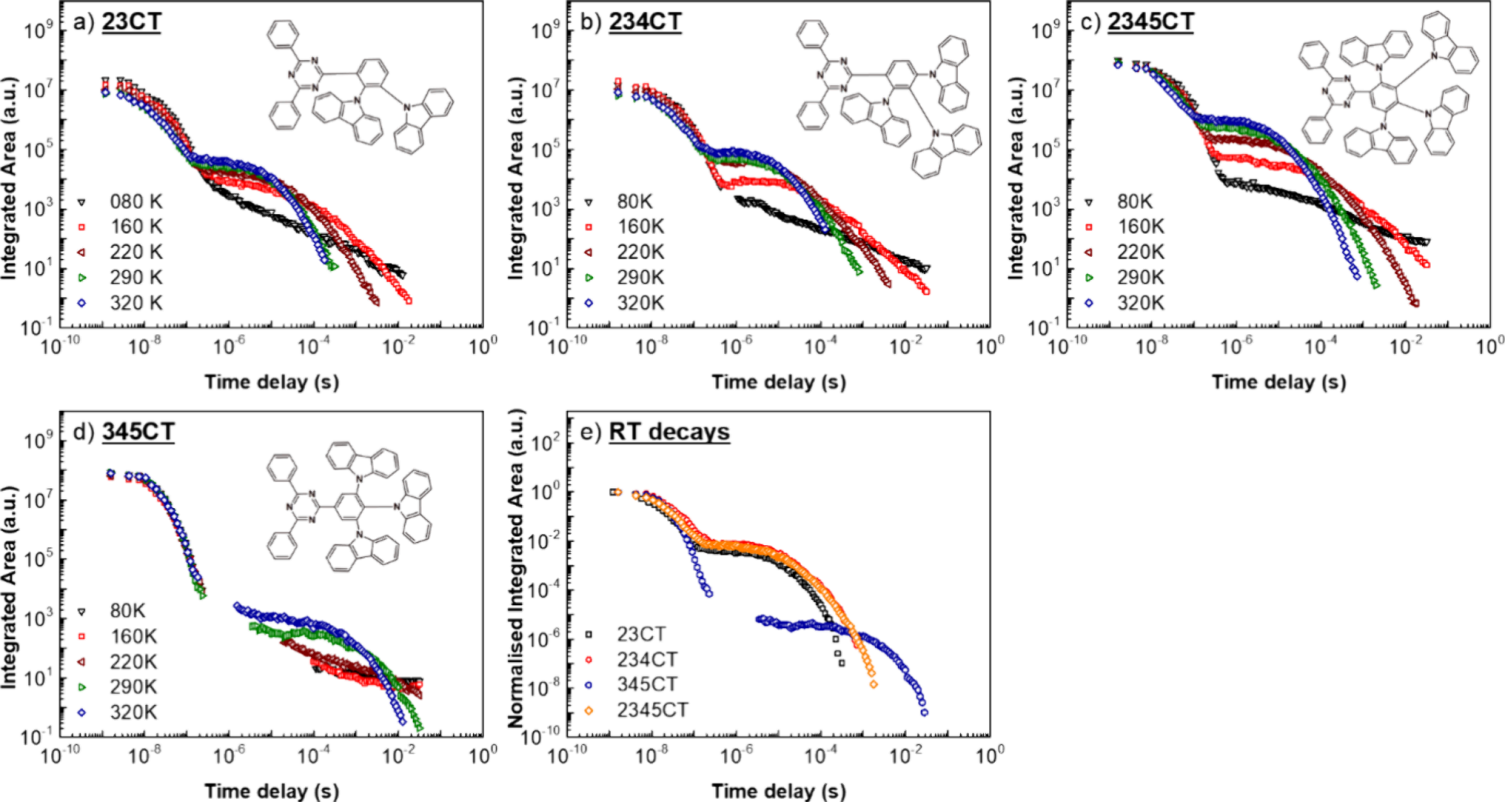
Time-resolved fluorescence decay curves at different temperatures of the four emitters in zeonex: (a) **23CT**, (b) **234CT**, (c) **2345CT**, and (d) **345CT**. In (e), the normalized decays of each at room temperature (RT) are overlapped. The molecular structure of each isomer is seen in the inset of each graph. Note gaps between data points (points not shown) in the decay curves are due to the signal detected being below the noise floor of the detector for specific time delays and integration times.

Figure 3 shows the time-dependent emission profiles of (a) **23CT**, (b) **234CT**, (c) **2345CT** and (d) **345CT** in the nonpolar zeonex matrix where emission from early (time delay–TD of 1.6 ns) to late times (TD up to 0.1 s) was collected between 80 and 320 K. In Figure 3e the normalized room temperature (RT) decays of all four are combined for better comparison.

Overall, all four emitters show two individual regions, PF (fast emission) and DF (long-lived emission), as the intensity of the DF component increases about 2 orders of magnitude with the increase in temperature. In the -*ortho* emitters, the DF decays are very similar (Figure 3e), although the saturation of the activated DF at higher temperatures decreases with increasing number of **D**s, i.e., **23CT** shows little increase of DF from 160 to 320 K, **234CT** starts to saturate at 220 K whereas **2345CT** only shows saturation at 290 K, indicative of decreasing ^3^CT-^3^LE coupling strength.^35^ The TADF origin of the DF in all materials was verified through analyses of emission intensity as a function of excitation power (Additional photophysical results shown in Figure S4) including **345CT,** which shows much slower DF.

**Figure 4:**
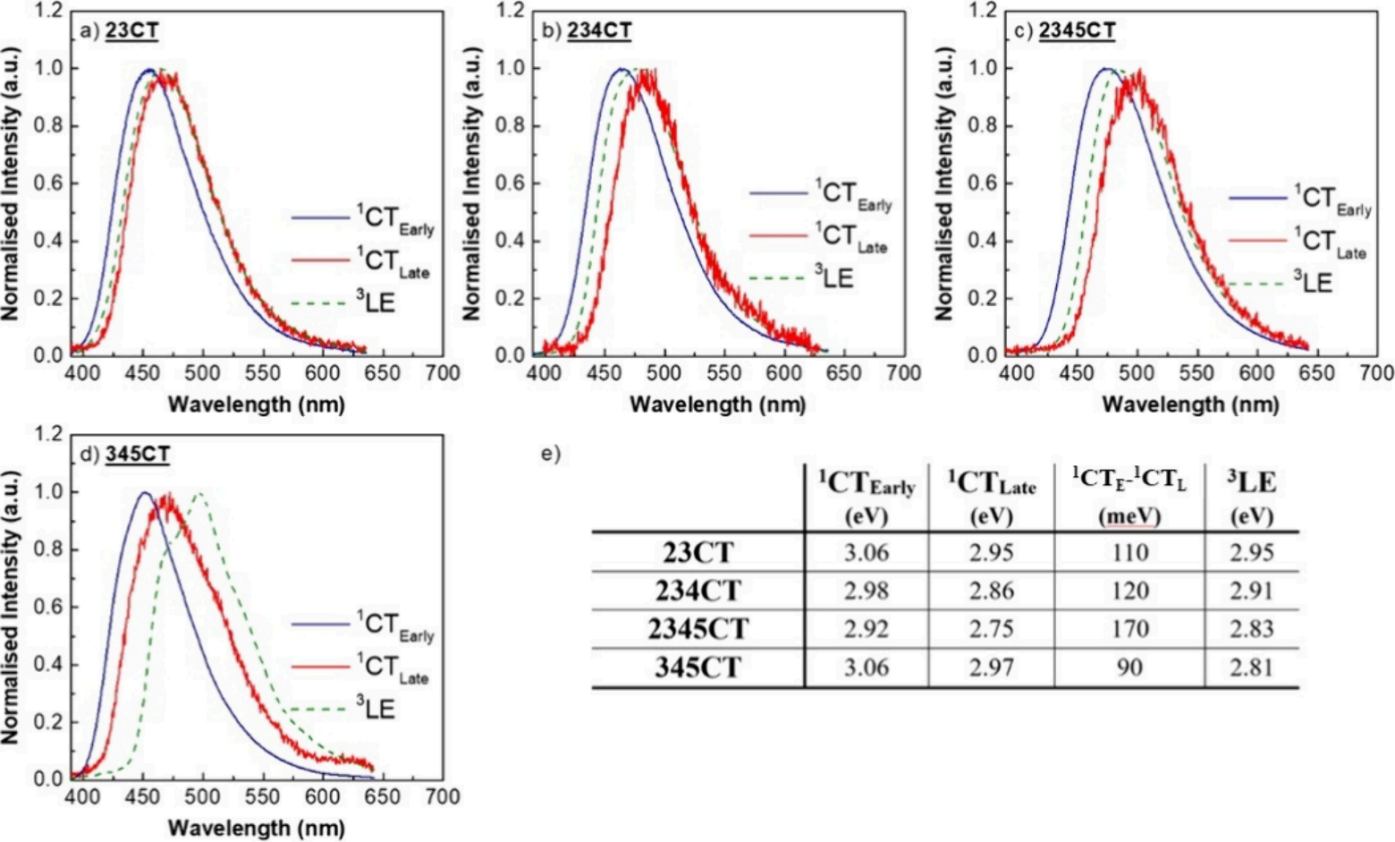
Energy levels of the emitters studied in a zeonex matrix: (a) **23CT**, (b) **234CT**, (c) **2345CT** and (d) **345CT**. The spectra were taken at 80 K with an excitation of 355 nm with different delay times: ^1^CT_Early_–1.6 ns; ^1^CT_Late_–100 to 300 ns; ^3^LE–above 25 ms. The onset of each spectrum provides the energy of the correspondent state, and the difference between ^1^CT_Early_ and ^1^CT_Late_ gives the width of the charge-transfer, ΔCT (e). Onset measurement with an error of ±0.02 eV.

Several works showed that the prompt CT emission of TADF molecules undergoes apparent dynamic redshift over the first few hundred nanoseconds which we explained as arising from heterogeneity of dihedral angles giving rise to a dispersion in CT energy and radiative decay rate.^4,14,36^ The most planar **D-A** has the weakest CT and the strongest excited state coupling to the ground state, so it emits in the blue with a faster radiative rate. The most orthogonal **D-A** on the other hand, has the strongest CT and therefore emits in the red with the slowest radiative rate.^34,36^ We recently reported a new approach to identify these distributions of D–A dihedral angles, by using an inverse Laplace transform fitting of delayed fluorescence of TADF molecules.^37^ Here, to represent this system’s (energy) dispersive ensemble of CT states, we measured the earliest prompt CT emission available, ^1^CT_Early_, collected at TD = 1.6 ns and the latest prompt CT emission, ^1^CT_Late_ collected at 100 ≤ TD ≤ 300 ns; Figure 4. With increasing number of **D**s, there is more dihedral inhomogeneity and thus the dispersion in CT energies broadens, from 110 meV FWHM in **23CT** to 170 meV FWHM in **2345CT**.

For all emitters at 80 K, emission collected at times longer than 25 ms was assigned as phosphorescence (PH) from local carbazole-like triplet states.^20^ PH from the **A** is found at ca. 3.1 eV for pure TRZ (additional photophysical results are shown in Figure S5), where there is no torsion around the acceptor; we note that in all -*ortho* emitters, one phenyl ring of the TRZ is twisted by around 40° which will localize the triplet state further, increasing its energy. Hence, the **A**^3^LE should play no part in the rISC of these molecules. For all *-ortho* molecules, the PH is Gaussian like and lies energetically within the dispersion in CT energies (Figure 4). This means that, there is a subset of CT and ^3^LE states having near zero energy gap, fundamentally yielding very high efficiency TADF.^31^ On the other hand, **345CT** shows a clear and resolved PH, lying energetically lower than the CT states (both early and late), which directly correlates with the poorer TADF performance. This PH has a similar shape to our previous findings on other reports on **D-A** systems based on carbazole and triazine **D** and **A**, respectively.^20,38^ Because of the long phosphorescence lifetime, as expected, we observe emission from only the lowest energy local triplet state in all cases.

By comparing the ΔCT (^1^CT_Early_ – ^1^CT_Late_), sampling blue to red emissive CT states, the most red emitters, i.e., the most orthogonal **D-A**, should give the fastest rISC as these should have the smallest ^1^CT-^3^CT gaps. However, the ^1^CT-^3^LE gap is the most important for rISC^14,35^ and the most orthogonal states will have the weakest radiative decay rate as well, thus it is not simple to correlate TADF efficiency with different CT states energies.

Looking at the decay rates and comparing them to the dispersion in CT energies may help us gain a better understanding of the decay channels involved in this family. From the PF and DF decay, we calculated the decay rates and rate constants of the intersystem crossing (*k*_ISC_) and reverse intersystem crossing (*k*_rISC_) processes, as shown in Figure S6.^20,39^ In all emitters containing carbazole in the *-ortho* position, with an increasing number of **D**s, the triplet yield increases, from 80% (**23CT**) to 84% (**234CT**) and 87% (**2345CT**). Fitting of the DF region can be complex, with many interacting channels occurring at the same time.^10,37^ Therefore, the number of exponentials and the weight of each exponential were both considered to achieve the best possible fitting. With this in mind, we see that the fitting of the TADF region becomes more complicated, transitioning from a monoexponential decay in **23CT** to a biexponential decay in **234CT** and a triple exponential decay in **2345CT**. We believe this reflects the number of nonequivalent **D-A** dihedral angles in each molecule as **D**s with similar angles can essentially be thought of as identical (Figure 2). Taking into consideration the angles calculated in the excited state S_1_, **23CT** exhibits two **D** units with comparable angles (82°), resulting in the observation of a single exponential decay. In the case of **234CT**, there are two D units with angles of ∼75° and one unit with an angle at 70°, leading to the detection of two exponential decays. Lastly, **2345CT** features two **D** units with an angle of ∼69°, one unit with an angle at 72°, and another unit with an angle at 64°, resulting in the detection of three distinct exponential decays. We can assume that the fastest channels (*k*_rISC__1) come from the more orthogonal carbazoles (which are rotated at 82, 75, and 72° for **23CT**, **234CT** and **2345CT**, respectively) as they have the smallest Δ*E*_ST_ and show the fastest τ_DF_. By adding more **D**s, with lower dihedral angles, slower components also start contributing strongly to both **234CT** and **2345CT**. Moreover, comparing the pre-exponential fitting factors of these slow components in **234CT** and **2345CT**, they constitute 24 and 79% of the DF contribution respectively, i.e., these longer lived species become more probable. **345CT** can be fitted with a biexponential decay dominated by a very slow rISC component with a 55% contribution in line with its large Δ*E*_ST_ due to the planarization of the molecule.

Therefore, each of these distinct dihedral angles will give rise to an associated distinct lowest ^3^LE state, having different energies coupling to different CT states with varying rISC rates, dependent on the associated energy gaps. With an increasing number of **D**s, this gives rise to an increasing number of rISC rates, and hence, more exponential fitting factors are required. These additional slow decay channels act as parasitic traps for the rISC decreasing the overall up-conversion rate back to the singlet state. The differences in the DF performance are only minimal, as the energy gaps are still small to provide efficient TADF and fast enough (overall) rISC rates. These slower rISC channels will, however, become more important in device operation. See Table 1 for time constants and decay rates of **23CT**, **234CT**, **2345CT**, and **345CT** in a zeonex matrix.

**Table 1:** Time Constants and Decay Rates of **23CT**, **234CT**, **2345CT**, and **345CT** in a Zeonex Matrix^a^

	τ_PF_ (ns)	τ_DF_1_ (μs)	τ_DF_2_ (μs)	τ_DF_3_ (μs)	ΦDF/ΦPF	ΦISC (%)	*k*_ISC_ (s^–1^)	*k*_rISC_1_ (s^–1^)	*k*_rISC_2_ (s^–1^)	*k*_rISC_3_ (s^–1^)
**23 CT**	8.4 ± 0.8	8.7 ± 0.3			4.01	80	9.53 × 10^7^	5.8 × 10^5^		
**234 CT**	19.6 ± 1.1	7.5 ± 0.8 (A1 = 17,339)	34.3 ± 11.4 (A2 = 5,411)		5.24	84	4.29 × 10^7^	8.32 × 10^5^	1.82 × 10^5^	
**2345 CT**	13.4 ± 0.6	1.1 ± 0.4 (A1 = 124,775)	9.3 ± 2.1 (A2 = 374,399)	44.5 ± 23.5 (A3 = 88,209)	6.46	87	6.49 × 10^7^		8.02 × 10^5^	1.66 × 10^5^
**345 CT**	14.6 ± 0.5	3.8 ± 0.7 (A1 = 249)	869.2 ± 72.3 (A2 = 315)		0.35^b^	26	1.78 × 10^7^	3.55 × 10^5^	1.55 × 10^3^	

a In the DF region, the best fitting was chosen and a correlation made to the photophysics. 23CT fitted best with a mono-exponential decay, **234CT** and **345CT** with a biexponential decay, and **2345CT** with a tri-exponential decay. A_n_ are pre exponential factors. More details on the calculation of these constants can be found in the literature.^20^

b It is generally accepted that if ΦDF/ΦPF is above 4, the product Φ_ISC_ Φ_rISC_ will be above 0.8. **345CT** does not fall in this category though the same assumption was applied for comparison purposes.

#### 2.2.1 Photophysics in DPEPO

The photophysical study so far shows a complex picture with multiple rISC channels and large energy distributions of the CT states. To be able to compare these results with device data, we also studied the complete photophysics of all the emitters in the host used in the device studies, namely, bis[2-(diphenylphosphino)phenyl]ether oxide (**DPEPO**).

**Figure 5:**
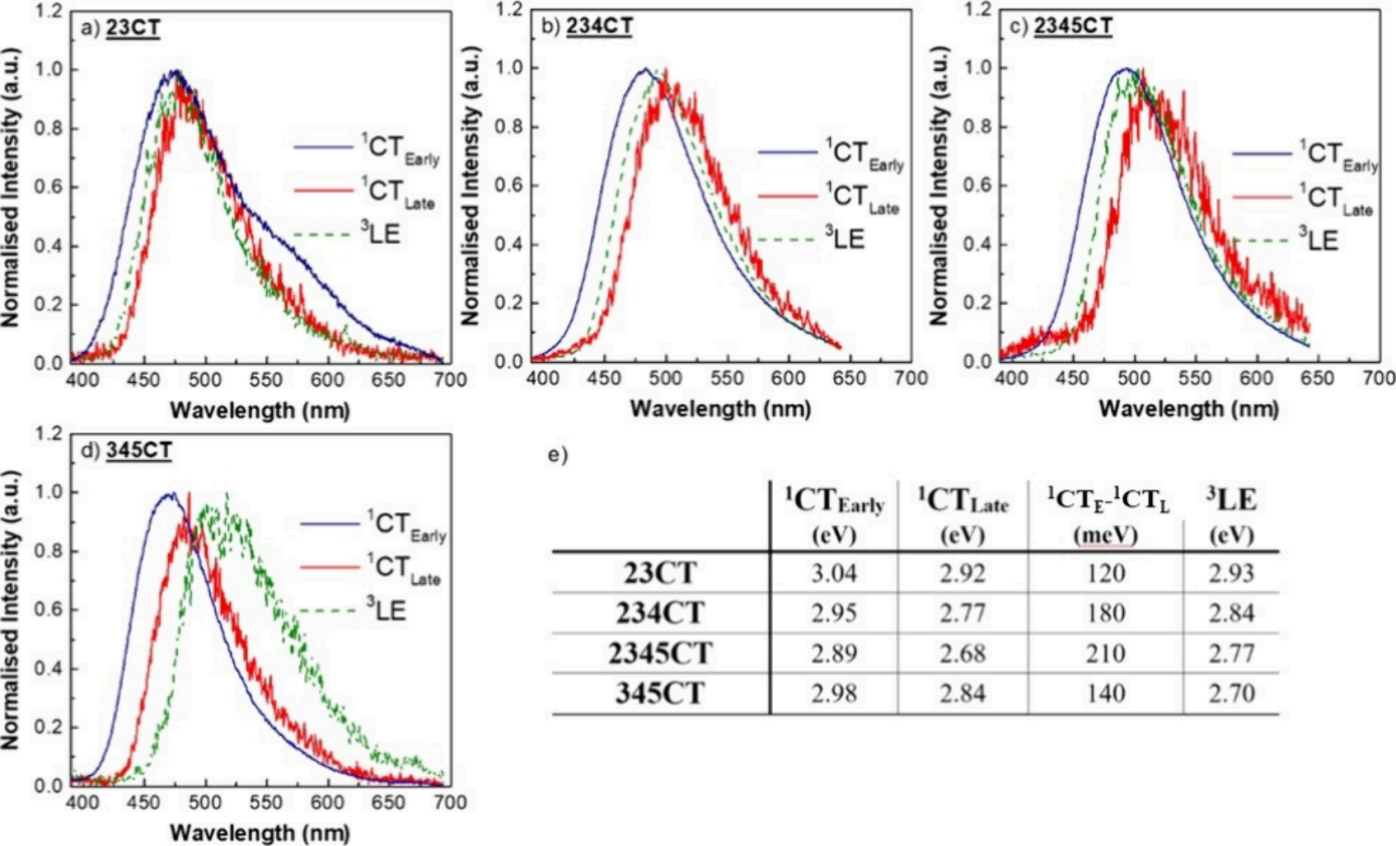
Energy levels of the emitters studied in a DPEPO matrix: (a) **23CT**, (b) **234CT**, (c) **2345CT**, and (d) **345CT** at 80 K and excited at 355 nm with different delay times. The onset of each spectrum provides the energy of the correspondent state, and the difference between ^1^CT_Early_ and ^1^CT_Late_ gives the width of the charge transfer, ΔCT.

Whereas the overall behavior of the emission (Figure 5) remains the same as in zeonex, two facts become immediately obvious: (i) the dispersion in CT energies broadens in **DPEPO**, and (ii) all PH spectra are red-shifted compared to zeonex (for comparison, see additional photophysical results shown in Figure S7). To rule out the possibility that the PH shift is dielectric dependent (DPEPO having a higher dielectric coefficient than zeonex), we measured the PH of **345CT** in another polymeric matrix with a higher dielectric strength than zeonex (PMMA) and confirmed that the onset does not change; see additional photophysical results shown in Figure S8. Therefore, we conclude that the redshift is an effect of the rigid host matrix packing, affecting the dihedral angles of the molecules and their conjugation, changing the energy gaps of the system, the formation of CT states at different energies, and especially the energies of the ^3^LE states. The relaxation of the CT energies must be through a combination of this packing effect and the dielectric strength of the host, however it should be noted that a relaxation of the excited-state energy through dielectric strength requires that the surrounding dielectric molecules reorient to stabilize the dipole moment of the CT molecule, and that such “solid-state” solvatochromism is greatly hindered in a dense host.^34,40^ In this sense, we believe the major effect is of the packing affecting the dihedral angles.

The decay curves at RT in **DPEPO** remain similar for **23CT** and **234CT** (Additional photophysical results shown in Figure S9) and we see a slight improvement in **345CT**. **2345CT** showed faster DF in **DPEPO** compared to zeonex; however, it still fit far better with a triexponential fitting which indicates that the slow parasitic rISC components are still present. Full fitting data are given in Table 2.

**Table 2:** Time Constants and Decay Rates of **23CT**, **234CT**, **2345CT**, and **345CT** in a DPEPO Matrix^a^

	τ_PF_ (ns)	τ_DF_1_ (μs)	τ_DF_2_ (μs)	τ_DF_3_ (μs)	ΦDF/ΦPF	ΦISC (%)	*k*_ISC_ (s^–1^)	*k*_rISC_1_ (s^–1^)	*k*_rISC_2_ (s^–1^)	*k*_rISC_3_ (s^–1^)
**23 CT**	16.2 ± 1.2	6.8 ± 0.3			3.93	80	4.9 × 10^7^	7.3 × 10^5^		
**234 CT**	27.2 ± 1.4	3.7 ± 0.4 (A1 = 99,400)	21.4 ± 4.3 (A2 = 43,700)		4.86	83	3.1 × 10^7^	1.4 × 10^6^	2.3 × 10^5^	
**2345 CT**	15.7 ± 0.8	0.3 ± 0.2 (A1 = 8431)	2.8 ± 0.5 (A2 = 67,900)	12.7 ± 3.6 (A3 = 21,000)	2.55^b^	72	4.6 × 10^7^	1.7 × 10^7^	1.8 × 10^6^	3.9 × 10^5^
**345 CT**	16.2 ± 0.5	3.8 ± 0.7 (A1 = 311)	869.2 ± 72.3 (A2 = 308)		0.67^b^	40	42.4 × 10^7^	9.0 × 10^5^	2.7 × 10^4^	

a *A*_n_ are pre exponential factors. More details on the calculation of these constants can be found in the literature.^20^

b It is generally accepted that if ΦDF/ΦPF is above 4, the product Φ_ISC_ Φ_rISC_ will be above 0.8. **2345CT** and **345CT** do not fall in this category though the same assumption was applied for comparison purposes.

From the photophysics measured in a dense rigid matrix (**DPEPO**) we can clearly see that the dihedral angles of each emitter are directly affected by the host. The rigidity of the host “locks in” the geometric structure of the molecule during film formation, and there is little chance for dihedral angles to equilibrate before the surrounding host molecules “freeze in” the structure. This translates into an increased width in the dispersion in CT energies for all the emitters (increased ΔCT), broadening with the number of **D**s. We can conclude that the host packing compresses the dihedral angles, increasing^41^ planarity. However, the lowering of the CT energies in DPEPO is matched by the lowering of the local triplet energies, and so the rISC rates and rISC channels remain rather unaffected compared to zeonex. Only **345CT** shows a larger difference; however, the gap is already large and undesirable.

### 2.3 Device Performance

**Figure 6:**
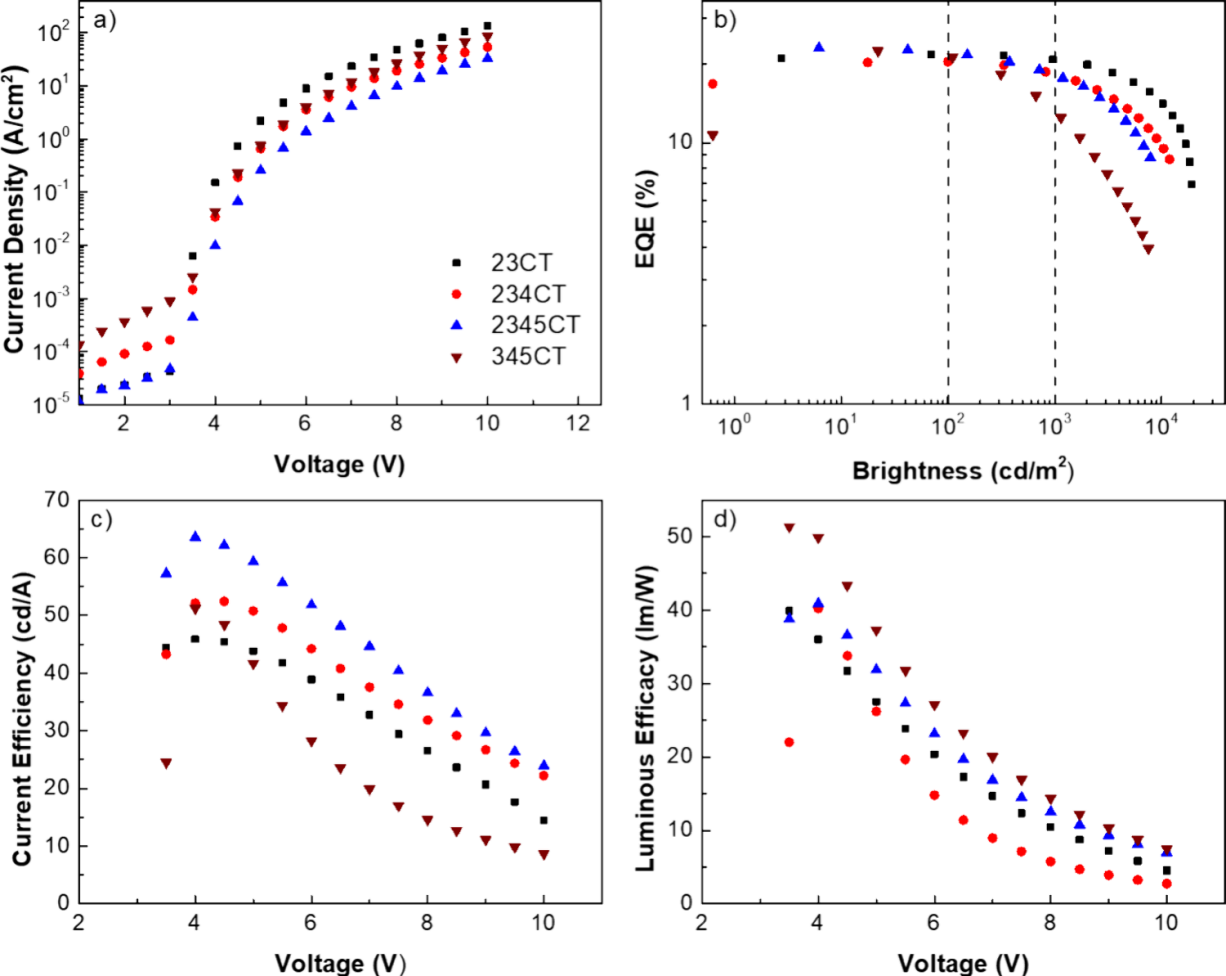
Characterization of devices based on **23CT**, **234CT**, **2345CT**, and **345CT**. (a) Current Density (*J*) over voltage; (b) external quantum efficiency (EQE) over brightness; (c) current efficiency over voltage; and (d) luminous efficacy over voltage.

Device performance of **2345CT** in a similar device structure to the one used in **23CT**,^20^**234CT,**^21^ and **345CT**(30) with **DPEPO** as a host is summarized in Table 3. All device electrical characterization is given in Figure 6.

**Table 3:** External Quantum Efficiency (η_ext_) of Devices Based on **23CT**, **234CT**, **2345CT**, and **345CT** and Commission Internationale de l’éclairage (CIE) at the Maximum Values, at 100 and 1000 cd/m^2^

	host	η_ext, max_ (%)	η_ext,100cd/m^2^_(%)	η_ext,1000cd/m^2^_ (%)	η_ext, drop_^a^ (%)	CIE (*x*, *y*)
**23CT**	DPEPO	21.8	21.8	20.8	4.6	(0.17, 0.33)
**234CT**		20.4	20.3	18.4	9.8	(0.20, 0.44)
**2345CT**		23.1	22.2	18.3	20.8	(0.22, 0.48)
**345CT**		22.4	21.2	13.3	40.6	(0.18, 0.37)

a Percentage drop between the maximum and 1000 cd/m^2^.

Overall, all devices showed high peak device efficiencies above 20%. Assuming an outcoupling efficiency between 20 and 30%, we believe this device stack is already optimized for its maximum performance.^42^ However, with an increasing number of **D**s, the roll-off starts increasing, going from a η_ext_ reduction of 4.6% in **23CT** to 9.8% in **234CT** and 20.8% in **2345CT,** between the maximum and at 1,000 cd/m^2^. **2345CT** also has the highest maximum η_ext_. The emission changes from sky-blue to greenish-blue, as a result of the change in CT emission (Figure 4). Similarly, **345CT**, shows the biggest η_ext_ reduction, 40.6%, the worst roll-off of the four, but still high peak η_ext_ and a sky-blue emission. All emitters have shown a linear dependence of the intensity with power (Figure S4) confirming the monomolecular character of the DF, and, thus, we believe that there is a strong correlation between roll-off and the increasing number of competing rISC components. This correlation comes in the form of an increasing probability of annihilation mechanisms like TTA and/or TPQ. In an optimized scenario, the **D-A** moieties would lock at the same ideal orthogonal angle, creating a single fast rISC channel. At high device current densities, this would minimize the presence of long-lived triplet states, resulting in excellent roll-off resistance with fast enough rISC. Our calculations and photophysics data show that by adding more **D** units (**2345CT**), we add slower rISC channels in the molecule, increasing the population of the long-lived triplet states and therefore increasing the probability of annihilation at high current densities. The increased weight of these slower channels (comparing **234CT** with **2345CT**) will increase the residence time of triplet states, making nonradiative transitions and TTA/TPQ more probable, especially as the density of triplets and charges increase. Therefore, we can predict device roll-off performance based on photophysical data by analyzing the dispersion of CT state energies which are directly related to dihedral angle. Through the large heterogeneity of dihedral angles due to multiple interacting **D**s and a rigid host matrix, the CT dispersion becomes very large. This allows more than one, nonequivalent ^3^LE state to couple with CT states with small enough energy gaps to yield an effective rISC rate.^20^ Increasing the number of **D**s increases the number of nonequivalent ^3^LE states and the number of rISC channels, concomitant with the number of different **D-A** dihedral angles.^10,43^ The different rates of each of these channels should be calculated through the vibronic coupling model.^4^ The fast rISC channel gives rise to the high maximum η_ext_ in devices and the rapid DF decay components in time-resolved photophysics. The slow rISC channels will increase the residence time of triplet states, making nonradiative transitions and TTA/TPQ more probably, especially as the density of triplets and charges increase, resulting in worse roll-off. This explains how the same molecule can give the highest maximum η_ext_ and worst roll-off performance at the same time, while another in the same family can give near η_ext,max_ but a much higher resistance to efficiency roll-off.

Moreover, our analyses connect well to other work, such as Wu et al., where the measured rISC rates are averages masking multiple contributions.^18^ The emission bands of Wu’s emitters are very broad and can very easily support multiple rISC modes coming from nonidentical **D-A** dihedral angles. Similarly, Chen’s systems^44^ all have roughly the same average DF decay times, which can mask multiple rISC channels which can explain why the materials have different η_ext_and roll-off behavior, which gets worse with more **D**s on the emitter, as shown here. Looking further, we can also analyze the set of device results reported by Guo et al. where the emitter-host combination with the lowest PLQY and lowest average rISC rate gives higher η_ext_ but poor roll-off compared to a neat host-free structure.^45^ We further apply our analyses to the work from Yu et al., who found that emitters with known **D** conformations (termed axial and equatorial^46^) gave much worse roll-off performance than an emitter with a **D** that could not form these different conformers.^47^ Again, another example showing that different conformations yield different rISC rates, the slower conformations controlling roll-off. We highlight that a direct comparison of our results with previous literature is complicated because the majority of these TADF papers only quote the averaged delayed fluorescence lifetime and calculate the single average rISC rate using this averaged lifetime. This masks the effect of the large heterogeneity of dihedral angles observed with crowded multiple **D**s that results in distinct delayed fluorescence lifetimes and consequently distinct rISC rates, affecting photophysics and device performance. Also, these papers present only one spectrum of the prompt fluorescence, not the time evolution of prompt and delayed emission. Therefore, they fail to explain how this dispersion of CT states giving fast and slow rISC rates controls device performance, as we demonstrate here.

Thus, our analyses give a new perspective on understanding and designing multifunctional molecules and how to control multiple competing electronic processes.

Finally, we suggest that when designing principles rules for efficient TADF materials (systems with multiple **D**s or **A**s) one must reduce the steric dispersion of dihedral angles. A compromise between the number of units and the number of these species they give rise should be met as to reduce the number of interacting rISC channels. Therefore, a design to reduce roll-off is to have symmetric multiunit systems that avoid the equivalent sites, like D-A-D or D-A_3_, such as the molecule reported by dos Santos et al.,^10^ where very high η_ext_ and excellent roll-off behavior were found.

## 3 Conclusions

In conclusion, this work demonstrates a correlation between the photophysical performance of single-acceptor/multidonor emitters and their resistance to roll-off in devices. Carbazole units with similar dihedral angles in the excited state act as a single rISC channel. By increasing the number of donor carbazoles in a multidonor–acceptor TADF system we have identified that nonequivalent donors (**D**’s with different dihedral angles) give rise to local donor triplet states with different energies. Furthermore, this large heterogeneity of dihedral angles gives rise to a large dispersion in the CT energies. Because the CT dispersion is energetically broad, more than one nonequivalent ^3^LE state can vibronically couple to CT states, giving rise to more than one rISC channel with different rISC rates. From this work, we understand that the fastest “primary” rISC channel causes fast DF decay and high maximum η_ext_ in devices, whereas the slower rISC channels cause long DF decay tails and poorer resistance to efficiency roll-off in devices. Thus, we can explain how within the same TADF family of emitters all can have very high peak η_ext_ and simultaneously have poor roll-off behavior. We also show how a rigid host matrix broadens the CT dispersion through packing effects, causing an overall lowering of dihedral angles and hence the energies of both the CT states and local triplet states.

## 4 Experimental Section

### 4.1 General Information

Synthesis of 2345CT can be found in S1. 9H-Carbazole, trimethyl borate, and *n*-butyllithium (2.5M) were purchased from Sigma-Aldrich Co., 1-bromo-2,3,4,5-tetrafluorobenzene was supplied by Santa Cruz Biotechnology, Inc. Cesium carbonate, potassium carbonate, *N*,*N*-dimethylformamide (DMF), hydrochloric acid were supplied by Duksan Co. Tetrakis(triphenylphosphine)palladium(0), 2-Chloro-4,6-diphenyl-1,3,5-triazine were bought from P&H Co. Tetrahydrofuran (THF), methylene chloride (MC), *n*-hexane were purchased from Samchun Pure Chemical Co., Ltd. 9H-carbazole was purified through a recrystallization method in toluene. Other reagents were used without any purification.

The final compounds were purified by using sublimation. For intermediate and final compounds, ^1^H-nuclear magnetic resonance (NMR), ^13^C NMR and mass spectrometry of materials were measured by using AVNACE III HD (Bruker, 500 MHz) spectrometer and Advion, Expresion^L^ CMS spectrometer with APCI mode, respectively.

### 4.2 Photophysics

For absorption and photoluminescence (PL) studies, each emitter was dispersed into solutions of 10^–3^–10^–5^ M of methylcyclohexane (MCH), toluene, and dichloromethane (DCM). Solutions of 2-4-6-Triphenyl-1-3-5-triazine (TRZ) and carbazole in MCH were also prepared. For the solid-state measurements in zeonex, toluene solutions of each emitter (with concentrations of 1 mg/mL) and zeonex (with a concentration of 100 mg/mL) were blended on a ratio of 1:1 wt/wt and dropcasted (∼100 μL) at room temperature. For the photophysics in DPEPO, solutions of each emitter were prepared in chloroform with concentrations of 1 mg/mL of emitter and 10 mg/mL of DPEPO. For each blend, the resulting solutions were mixed in a 1:1 ratio and around 70 μL spin-coated onto a sapphire substrate, for 30 s and 2000 rpm using a Laurell Technologies spin-coater.

Absorption and emission spectra of both solution and solid-state samples were collected using a UV-3600 double beam spectrophotometer (Shimadzu) and Jobin Yvon Horiba Fluoromax 3. Time-resolved spectra were obtained by exciting the solid state samples with a Nd:YAG laser (EKSPLA), 10 Hz, 355/266 nm or by using a Nitrogen laser, 10 Hz, 337 nm. When necessary, the frequency of the laser was adjusted to determine emission at TD longer than 0.1 s. For the Nd:YAG laser and Nitrogen laser the earliest emission available for collection was at time delays (TD) of 1 and 30 ns, respectively. Sample emission was directed onto a spectrograph and a gated iCCD camera (Stanford Computer Optics). The rISC constant calculations were completed following the procedure in the literature.^20,39^ In the power dependence measurements, the intensity of the laser was varied from 70 to 0.1 μJ and the emission in the delayed region was collected.

### 4.3 Device Fabrication and Characterization

The devices were fabricated on an indium tin oxide (ITO) substrate (AMG Co.). Hole injection, hole transport, and electron blocking layers were composed of poly(3,4-ethylenedioxythiophene):poly(styrenesulfonate) (PEDOT:PSS), 4,4′-cyclohexylidenebis[*N*,*N*-bis(4-methylphenyl)benzenamine] (TAPC) and 1,3-bis(*N*-carbazolyl)benzene (mCP), respectively. For hole blocking and electron transport, diphenyl-4-triphenylsilylphenyl-phosphine oxide (TSPO1) and 2,2′,2″-(1,3,5-benzinetriyl)-tris(1-phenyl-1-benzimidazole) (TPBi), were used. The emitting layer was a coevaporation of 30 wt % 2345CT in bis[2-(diphenylphosphino)phenyl] ether oxide (DPEPO) layer and comparisons were made for the same device structure using the device performance obtained for emitters **23CT**,^20^**234CT**^21^, and **345CT**(30) in their respective papers. The overall device structure was ITO (120 nm)/ PEDOT:PSS (60 nm)/ TAPC (20 nm)/ mCP (10 nm)/ DPEPO:2345CT (25 nm, 30 wt %)/ TSPO1 (5 nm)/ TPBi (20 nm)/ LiF (1.5 nm)/ Al (200 nm).

Keithley 2400 electrical source unit and CS 1000 (Minolta Co.) optical measurement units were used for current density, luminance and electroluminescence spectrum by voltage sweep measurements.^37^

### 4.4 Computational Details

Computations were performed with the Gaussian 09 package^48^ using various density functional theory (DFT) methods. Ground state geometries of the studied compounds were accessed at the rCAM-B3LYP/6-31G(d) level of theory, while the S_1_ excited state configurations were obtained using Tamm-Dancoff approximation at the TDA-DFT CAM-B3LYP/6-31G(d) level. Multiwfn12 software was used to evaluate molecular fragment contributions to occupied and virtual orbitals. The TDA approximation was used in order to give a more accurate description of the CT transitions from our TD-DFT calculations.^49,50^

## Data Availability

All data used in this work are given in the main manuscript or in the Supporting Information.

## Supporting Information Available

Synthesis of 2345CT, additional computational results, additional photophysical results and decay rate calculations, materials and synthesis, and supplementary figures and discussion (PDF)
