# Supplementary material for: Influence of Multiple rISC Channels on the Maximum Efficiency and Roll-Off of TADF OLEDs
Source: J Phys Chem C Nanomater Interfaces. 2024 Sep 19;128(39):16308–19. doi: 10.1021/acs.jpcc.4c02993 (PMC11457217; doi:10.1021/acs.jpcc.4c02993)
Supplement: Supplementary file 1 — jp4c02993_si_001.pdf [file jp4c02993_si_001.pdf]

Supplementary Information

Influence of Multiple rISC Channels on the Maximum Efficiency and Roll-Off of TADF OLEDs

Paloma Lays dos Santos*[1], Daniel de Sa Pereira[2], Chan Seok Oh[3], Nadzeya Kukhta[4], Ha Lim Lee[3], Jun Yeob Lee*[3,5], Andrew P. Monkman*[2]

1 Department of Electronic and Electrical Engineering, University of Sheffield
Mappin St, Sheffield, S1 3JD, United Kingdom

2 Department of Physics, Durham University
South Road, Durham, DH1 3LE, United Kingdom

3 School of Chemical Engineering, Sungkyunkwan University
2066, Seobu-ro, Jangan-gu, Suwon, Gyeonggi, 16419, Korea

4 Department of Chemistry, Durham University
South Road, Durham, DH1 3LE, United Kingdom

5 SKKU Institute of Energy Science and Technology, Sungkyunkwan University
2066, Seobu-ro, Jangan-gu, Suwon, Gyeonggi, 16419, Korea

Figure S1. Synthesis of 2345CT

Apart from 2345CT, all other molecules used in this work were prepared following the synthetic procedure available: 23CT[1], 234CT[2] and 345CT[3]. The synthesis of 2345CT is shown next.

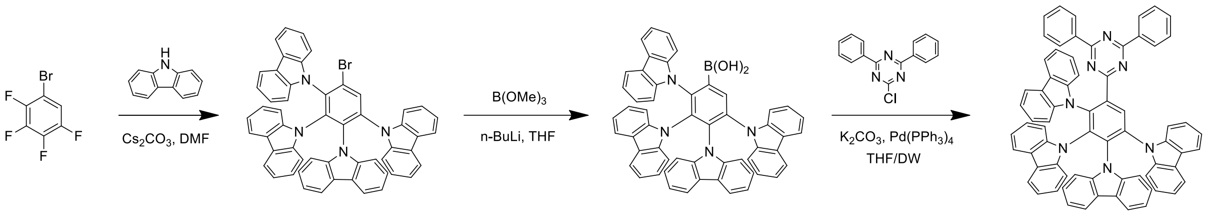

9,9',9'',9'''-(5-bromobenzene-1,2,3,4-tetrayl)tetrakis(9H-carbazole)

9H-Carbazole, trimethyl borate, n-butyllithium (2.5M) was purchased from Sigma-Aldrich Co., 1-bromo-2,3,4,5-tetrafluorobenzene was supplied by Santa Cruz Biotechnology, Inc.. Cesium carbonate, potassium carbonate, N,N-dimethylformamide (DMF), hydrochloric acid were supplied by Duksan Co. Tetrakis(triphenylphosphine)palladium(0), 2-Chloro-4,6-diphenyl-1,3,5-triazine were bought from P&H Co.. Tetrahydrofuran (THF), methylene chloride (MC), n-hexane were purchased from Samchun Pure Chemical Co., Ltd.. 9H-carbazole was purified through a recrystallization method in toluene. Other reagents were used without any purification.

1-Bromo-2,3,4,5-tetrafluorobenzene (1.00 g, 4.37 mmol), 9H-carbazole (3.29 g, 19.65 mmol), and cesium carbonate (7.11 g, 21.84 mmol) were mixed and dissolved in DMF (60 mL). The reaction mixture was then poured into a 200 mL pressure tube and stirred and heated with a reflux condition for 12 h. At the end of the reaction, the mixture was cooled down to room temperature. The crude solution was extracted using dichloromethane and the extracted organic layer was concentrated by evaporating the solvent. The concentrated mixture was adsorbed into silica gel and purified by column chromatography using a eluent solvent (MC : n-hexane = 1 : 2). A white solid product was obtained as a pure solid (2.54 g, 72% Yield). The final compounds were purified using sublimation.

MS (APCI) m/z : Found 819.25 [$(M + H)^+$]. Calculated For $C_{54}H_{33}BrN_4$: 817.77

9,9',9'',9'''-(5-(4,6-diphenyl-1,3,5-triazin-2-yl)benzene-1,2,3,4-tetrayl)tetrakis(9H

carbazole) (2345CT)

9,9',9'',9'''-(5-bromobenzene-1,2,3,4-tetrayl)tetrakis(9H-carbazole) (1.00 g, 1.22 mmol) was poured into a 100 mL, 2-neck round bottom flask and fully dried in vacuum for 24 hours. Then the reaction kit was connected with a nitrogen line and made inside of flask to be in a nitrogen atmosphere. THF (20 ml) was injected into a round bottom flask and the solution cooled down to -76°C using dry ice/acetone bath. After cooling for 1 h, n-butyllithium (0.80 mL, 1.83 mmol) was dropped slowly into the solution. Lithiation was proceeded for 1 hour and trimethyl borate (0.41 ml, 3.66 mmol) was dropped into the mixture. The reaction occurred for 24 hours and the mixture was quenched with 2M HCl solution (10 ml). After quenching, the reaction mixture was extracted with methylene chloride and filtered. The filtered product was a white powder (0.61 g, 64% Yield).

2-chloro-4,6-diphenyl-1,3,5-triazine (0.34 g, 1.28 mmol), (2,3,4,5-tetra(9H-carbazol-9-yl)phenyl)boronic acid (1.00 g, 1.28 mmol) were dissolved into THF (10 mL). The mixed solution was added slightly heated in a 2-neck round bottom flask. Then the 2M potassium carbonate solution was added into a reactant mixture, following addition of tetrakis(triphenylphosphine)palladium(0) (0.04 g, 0.04 mmol). The reaction solution was then stirred and refluxed overnight. Next the reaction mixture was cooled down and extracted with MC. The extracted organic layer was concentrated and adsorbed into silica gel. The crude product was purified using column chromatography with a mixed solvent (MC: n-hexane=1:1). The product was further purified by using train sublimation with 2 times. After purification, a bright yellowish green powder was obtained (0.73 g, 59% Yield).

MS (APCI) m/z : Found 971.31 $[(M + H)^+]$. Calculated For $C_{69}H_{43}N_7$: 970.13

Figure S2. Additional computational results.

Ground (rCAM-B3LYP/6-31G(d)) and excited (TDA-DFT CAM-B3LYP/6-31G(d)) states frontier orbitals (HOMO, LUMO) of the studied compounds.

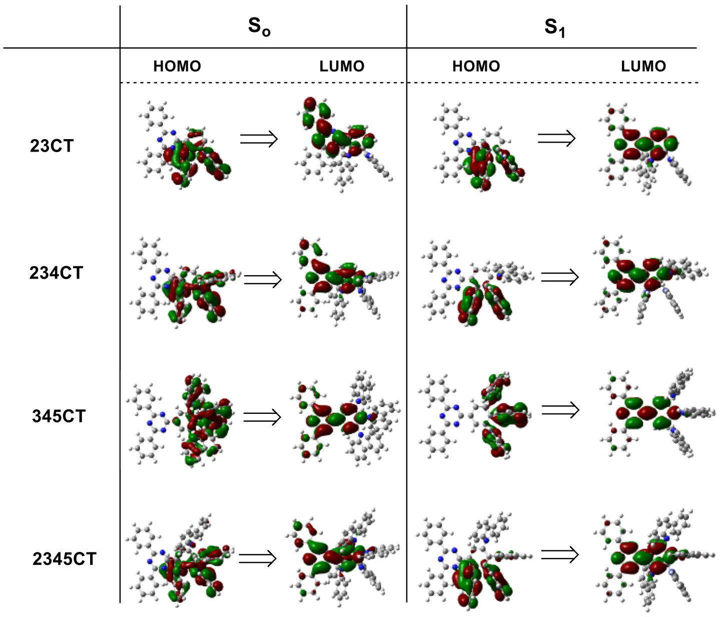

Figure S3. Additional photophysical results.

Photophysics of 23CT, 234CT, 2345CT and 345CT in solution. a)absorption in dimethylcyclohexane (DCM) with absorption of 2,4,6-triphenyl-1,3,5-triazine (TRZ) and carbazole as an inset. b) solvatochromism study of each emitter in solvents wih increasing polarity – methycyclohexane (MCH, dielectric constant, ε = 2.02), toluene (ε = 2.4) and DCM (ε = 9.0). In MCH, the emitters show vibronic-like features, which is associated with a higher local excited singlet (^{1}LE) character or the mixing of both ^{1}LE and ^{1}CT. In toluene and DCM, the emission spectra become gaussian-like and start to redshift.

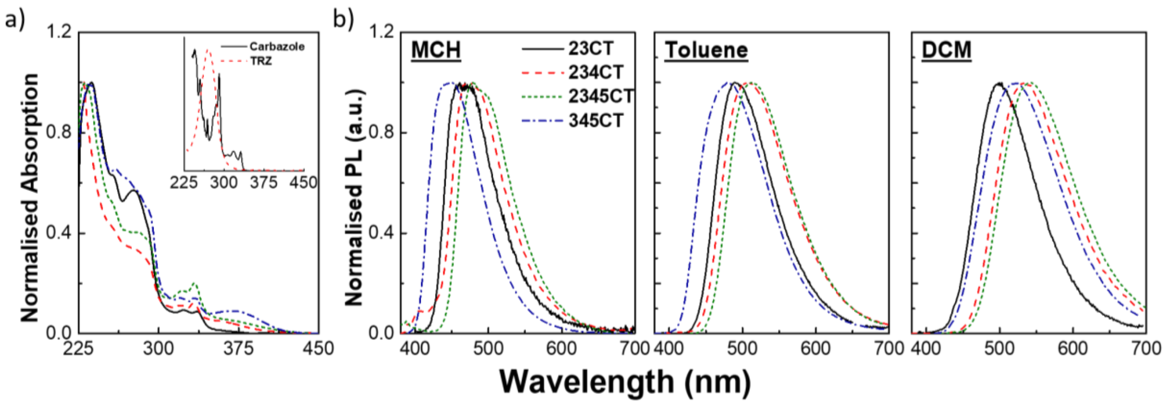

Figure S4. Additional photophysical results.

Power dependence of each emitter in zeonex at room temperature. Emission was collected at each delayed fluorescence region: a) 23CT (141 to 9062.9 ns); b) 234CT (300 to 44500 ns); c) 2345CT (200 to 6300 ns); d) 345CT (3.45 μs to 281.82 μs) and with power between 60 nJ to 80 μJ.

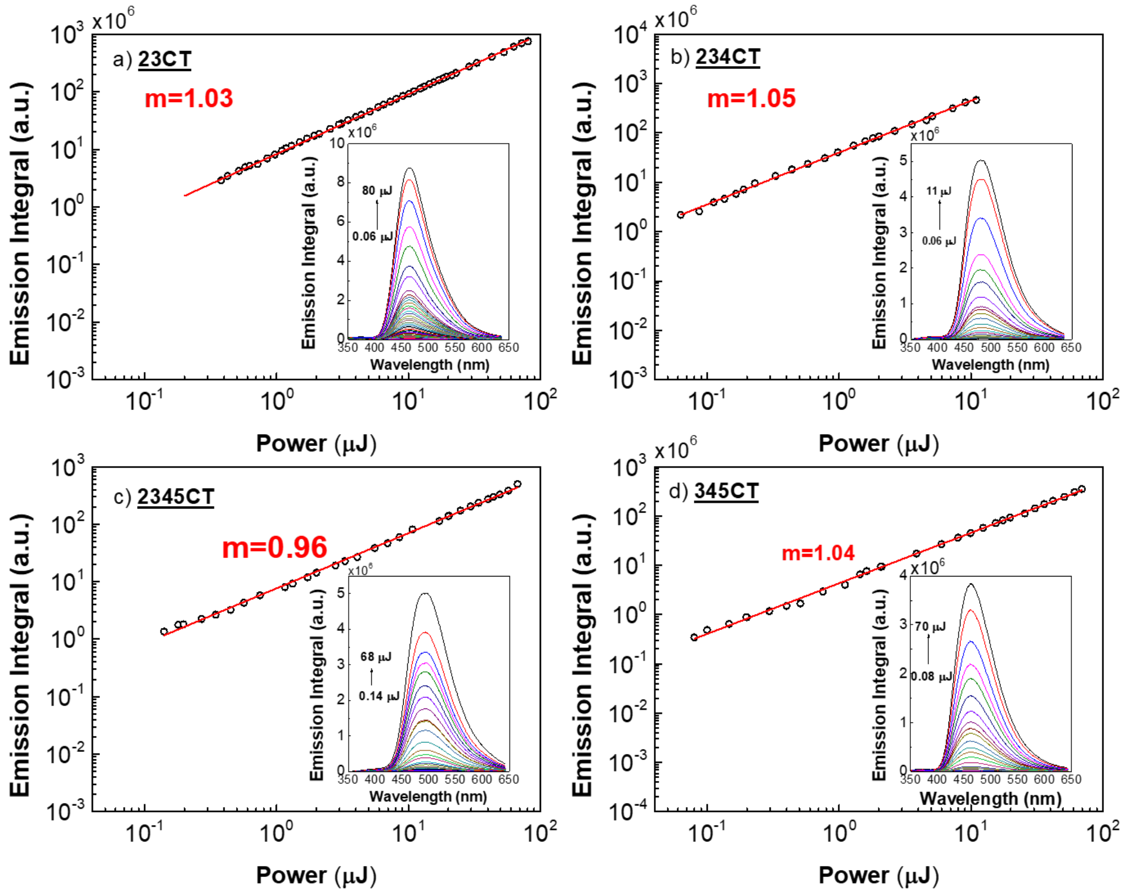

Figure S5. Additional photophysical results.

Normalised phosphorescence (PH) spectra of 2,4,6-triphenyl-1,3,5-triazine in a zeonex host. Onset energy gives a ^{3}LE state of 3.1 eV +/- 0.02 eV. PH collected with a time delay above 25 ms and at 80 K.

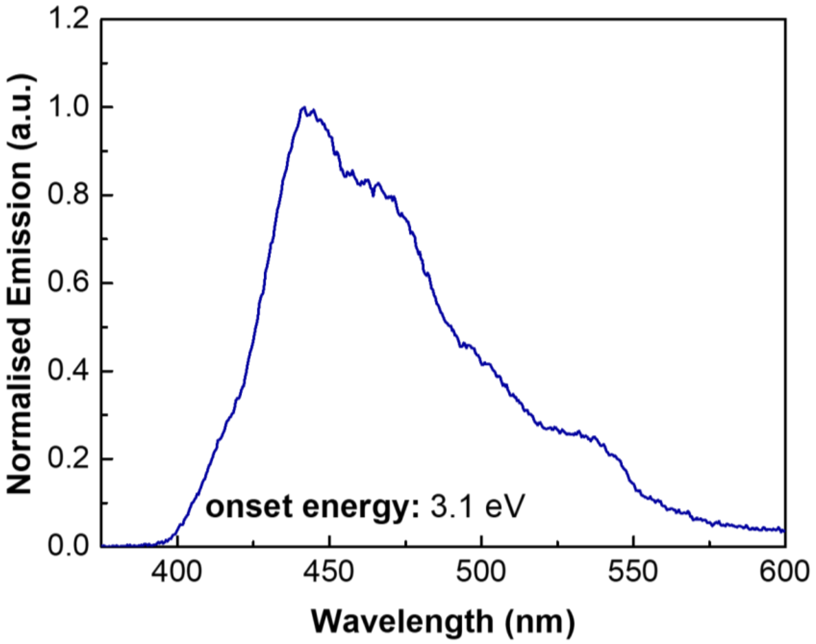

Figure S6. Decay rates calculations.

Decay rates of the prompt (τ_{PF} – A) and delayed (τ_{DF} – B) fluorescence of the emitters from this study, a) 23CT, b) 234CT, c) 2345CT and d) 345CT. The expression used in the single, double and triple exponential fittings is shown below. All fittings obtained are in nanoseconds. 23CT showed mono-exponential DF, 234CT and 345CT showed a bi-exponential DF decay and 2345CT a triple exponential decay.

Single Exponential Decay
$$y = y_0 + A_1 \exp\left(\frac{-(x - x_0)}{t_1}\right)$$
**Double Exponential Decay**
$$y = y_0 + A_1 \exp\left(\frac{-(x - x_0)}{t_1}\right) + A_2 \exp\left(\frac{-(x - x_0)}{t_2}\right)$$
**Triple Exponential Decay**
$$y = y_0 + A_1 \exp\left(\frac{-(x - x_0)}{t_1}\right) + A_2 \exp\left(\frac{-(x - x_0)}{t_2}\right) + A_3 \exp\left(\frac{-(x - x_0)}{t_3}\right)$$

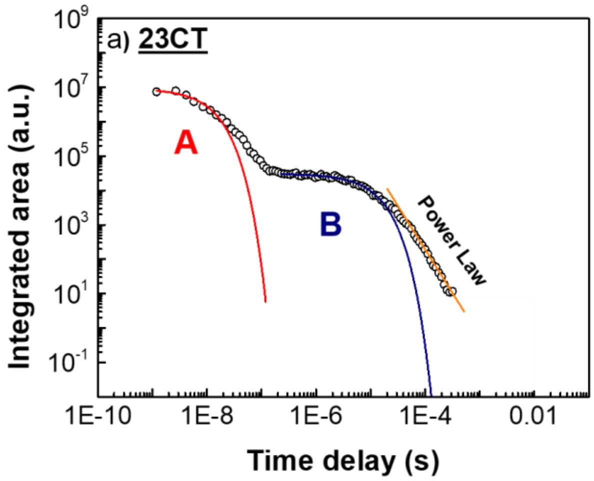

Model	A) Single Exponential Decay		
Reduced Chi – Sqr	2.35e11		
Adj. R. Square	0.96483		
		Value	Standard Error
CALC	y0	0	0
	X0	1.2	0
	A1	7.94795e6	365012.62
	t1 (ns)	8.40216	0.79121

Model	B) Single Exponential Decay		
Reduced Chi – Sqr	1.81e6		
Adj. R. Square	0.98692		
		Value	Standard Error
CALC	y0	0	0
	X0	234.25	0
	A1	29729.66	345.07
	t1 (ns)	8688.37	327.94

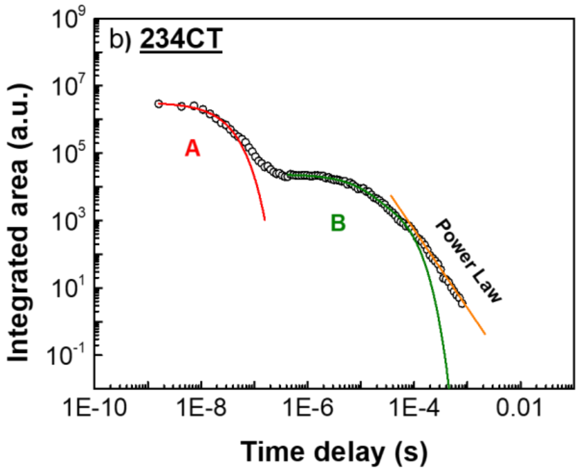

Model	A) Single Exponential Decay		
Reduced Chi – Sqr	1.49e10		
Adj. R. Square	0.98345		
		Value	Standard Error
CALC	y0	0	0
	X0	1.6	0
	A1	2.96e6	8.59e4
	t1 (ns)	19.61	1.10

Model	B) Double Exponential Decay		
Reduced Chi – Sqr	4.22e5		
Adj. R. Square	0.99446		
		Value	Standard Error
CALC	y0	0	0
	X0	425.9	0
	A1	17338.56	2116.13
	t1 (ns)	7510.67	886.64
	A2	5410.55	2175.26
	t2 (ns)	34332.50	11387.60

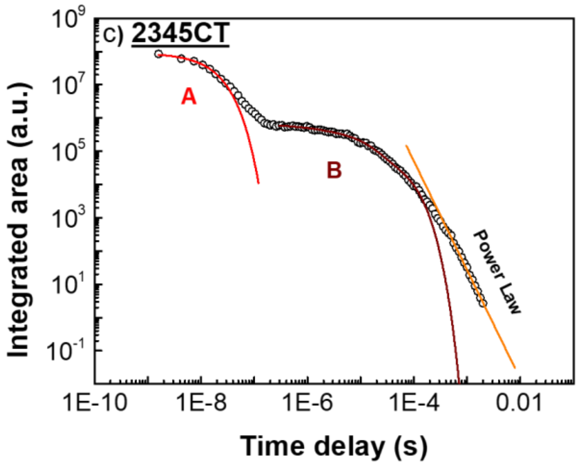

Model	A) Single Exponential Decay		
Reduced Chi – Sqr	5.13e12		
Adj. R. Square	0.99151		
		Value	Standard Error
CALC	y0	0	0
	X0	1.6	0
	A1	7.93e7	1.77e6
	t1 (ns)	13.40	0.57

Model	B) Triple Exponential Decay		
Reduced Chi – Sqr	2.30e8		
Adj. R. Square	0.99487		
		Value	Standard Error
CALC	y0	0	0
	X0	295.4	0
	A1	124775.19	29642.26
	t1 (ns)	1100.15	362.37
	A2	374399.07	46586.45
	t2 (ns)	9252.22	2090.79
	A3	88209.47	60021.35
	t3 (ns)	44489.71	23450.45

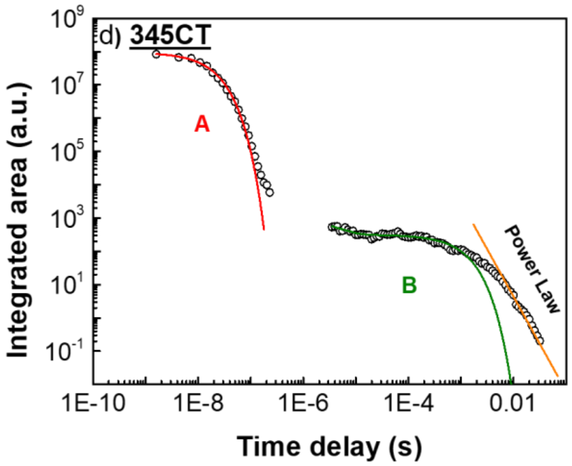

Model	A) Single Exponential Decay		
Reduced Chi – Sqr	4.02e12		
Adj. R. Square	0.99421		
CALC		Value	Standard Error
	y0	0	0
	X0	1.6	0
	A1	8.45e7	1.53e6
	t1 (ns)	14.62	0.50

Model	B) Double Exponential Decay		
Reduced Chi – Sqr	991.06		
Adj. R. Square	0.96053		
CALC		Value	Standard Error
	y0	0	0
	X0	3527.5	0
	A1	248.97	21.55
	t1 (ns)	3765.95	700.09
	A2	314.88	7.87
	t2 (ns)	869184.42	72327.99

Figure S7. Additional photophysical results.

Normalised phosphorescence (PH) spectra of a) 23CT, b) 234CT, c) 2345CT and d) 345CT in a zeonex matrix and in a DPEPO host. All PH were collected with a time delay above 25 ms and at 80 K to avoid contribution from any delayed singlet emission. Overall, the PH in DPEPO are more redshifted due to changes in the packing of the emitters induced by the host. One that affects the dihedral and torsional angles and in turn affect the conjugation of the molecules.

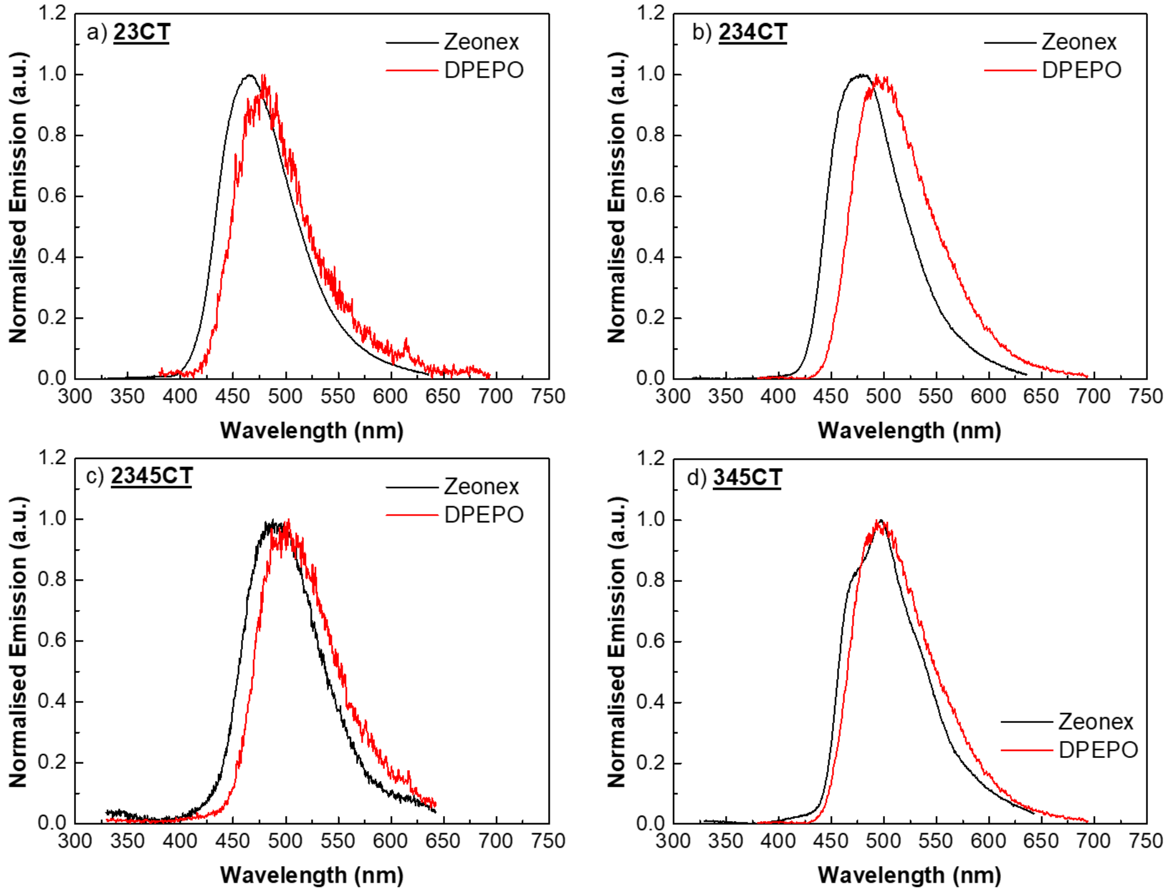

Figure S8. Additional photophysical results.

Normalised phosphorescence of 345CT in zeonex and in PMMA matrixes. All PH were collected with a time delay above 25 ms and at 80 K to avoid contribution from any delayed singlet emission. PMMA has a slightly higher polarity in comparison to zeonex. However, the emission of 345CT is completely overlapped in both hosts meaning that the redshift seen between zeonex and DPEPO is not polarity dependant.

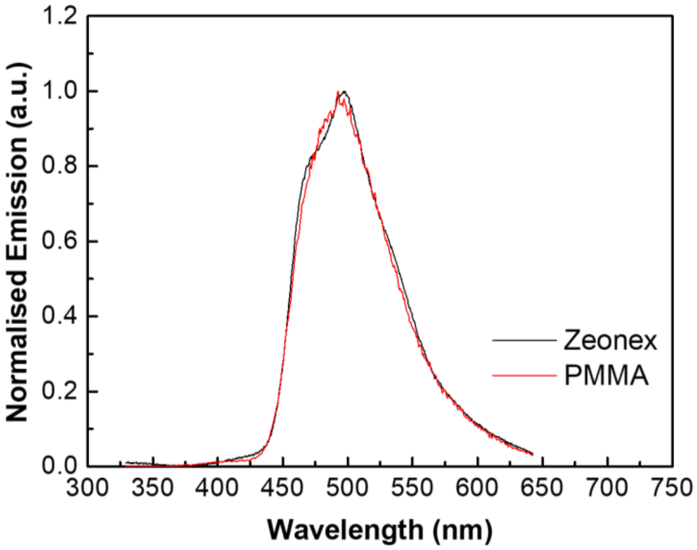

Figure S9. Additional photophysical results.

Normalised room temperature decays of a) 23CT, b) 234CT, c) 2345CT and d) 345CT in a zeonex matrix overlapped with their decays in DPEPO.

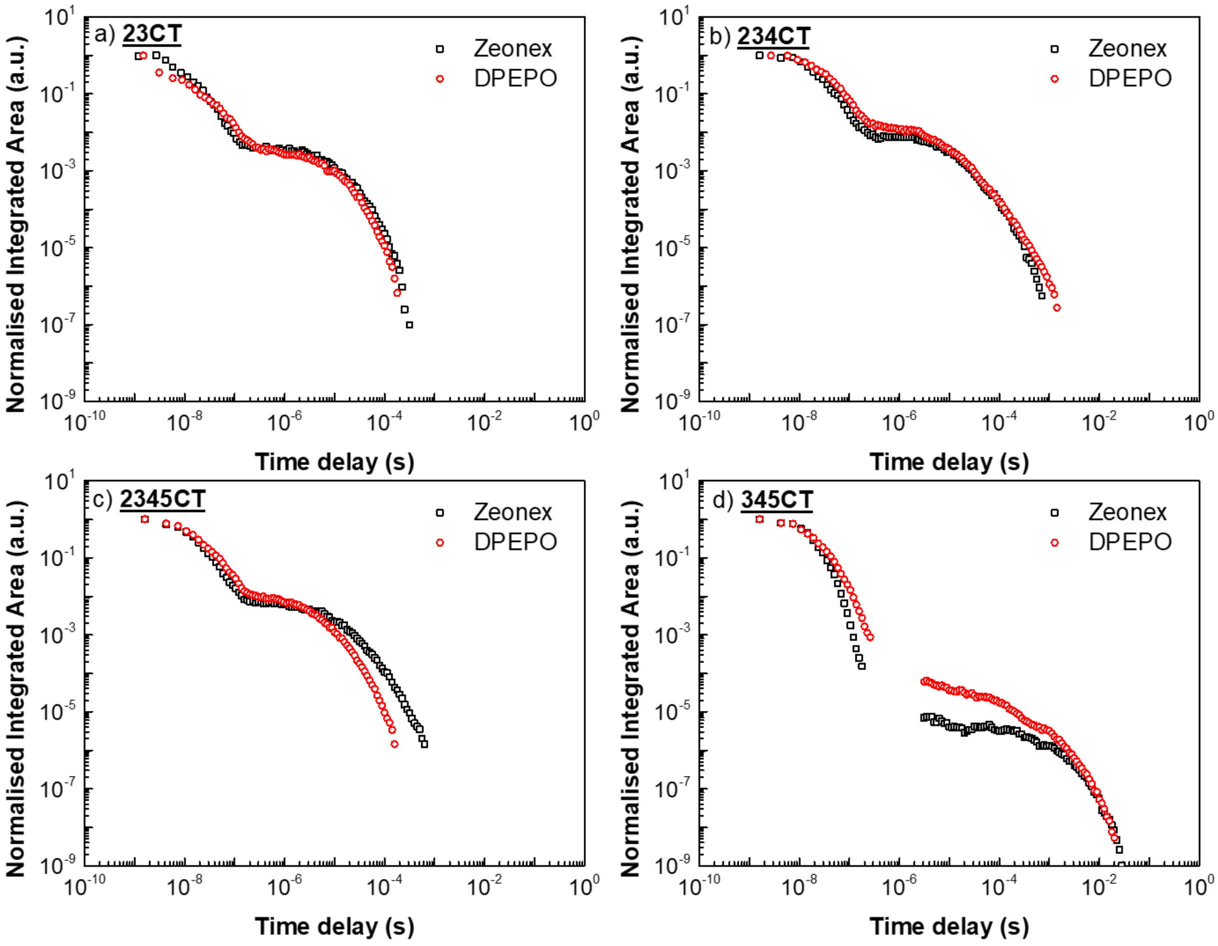

References

1. Oh, C. S. *et al.* Dihedral Angle Control of Blue Thermally Activated Delayed Fluorescent Emitters through Donor Substitution Position for Efficient Reverse Intersystem Crossing. *ACS Appl. Mater. Interfaces* **10,** 35420–35429 (2018).

2. Oh, C. S., Lee, H. L., Han, S. H. & Lee, J. Y. Rational molecular design overcoming the long delayed fluorescence lifetime and serious efficiency roll-off in blue thermally activated delayed fluorescent devices. *Chem. - A Eur. J.* **25,** 642–648 (2019).

3. Lee, D. R. *et al.* Design Strategy for 25% External Quantum Efficiency in Green and Blue Thermally Activated Delayed Fluorescent Devices. *Adv. Funct. Mater.* **27,** 5861–5867 (2015).
